# Understanding Complaints in the Emergency Department

**DOI:** 10.1177/11786329211057351

**Published:** 2021-12-06

**Authors:** Alina Abidova, Pedro Alcântara da Silva, Sérgio Moreira

**Affiliations:** 1 National School of Public Health, NOVA University of Lisbon, Lisbon, Portugal; 2 Institute of Social Sciences, University of Lisbon, Lisbon, Portugal; 3 Faculty of Psychology, University of Lisbon, Lisbon, Portugal

**Keywords:** Patient satisfaction, perceived quality of healthcare, emergency department, patients’ complaints, frequency of ED experiences, frequent users, waiting time for triage, privacy

## Abstract

The aim of this research is to identify the main determinants of patients’ complaints and potential mediators and moderators in this regard. This research shows that complaints can result from a complex set of processes involving direct, mediating, and moderating effects. Interventions aimed at reducing patients’ complaints should consider specific patient groups and experiences.

## Introduction

The number of emergency department (ED) visits is steadily increasing in most high-income countries.^1^ Researchers have emphasized several factors that contribute to ED visits: inability to cope with pain; fear; access to other services and resources; and suggestions from family to go to the ED.^2^

It is estimated that 1% to 5% of the entire ED population comprises frequent users.^3^ Patients who visit the ED frequently are a particularly vulnerable population group.^4^ When compared to infrequent users, frequent ED users are more likely to have chronic disease and poor physical health, resulting in higher hospital admission rates and mortality.^4^ Various thresholds for the definition of frequent ED users exist in the literature, with no consensus on a single definition (eg, 3-10 ED visits within 12 months,^3^ 4 or more ED visits,^5,6^ or 5 or more ED visits per year^7^).

Patients may return to the same medical facility repeatedly over time because they are satisfied with their experience; however, repeat/frequent visits may not necessarily indicate patient loyalty resulting from satisfaction.^8^ In some cases, patients cannot easily change the medical facility they visit and thus temporarily continue using the same provider despite an unsatisfactory experience.^8^

Additionally, patients become more informed about the health care services through frequent visits and can incorporate a wider set of factors into their service quality assessment.^8^ Therefore, patients may become more critical of professional practices through increased interactions.^8^

Patient satisfaction is associated with complaints and correlated with malpractice lawsuits (problems with patient care quality) and communication problems.^9^ In turn, patient complaints are associated with malpractice lawsuits.^10^

In this study, we sought to understand the determinants of patients’ complaints, whether the effect of the predictors (antecedents) on patients’ complaints is mediated by the perceived quality of healthcare (PQHC) and satisfaction, and whether the effect of PQHC and satisfaction on patients’ complaints is moderated by patients’ characteristics.

## Methods

Authorization for this study was obtained from the Ethics Committee and Board of Directors and Administration of the Centro Hospitalar de Lisboa Ocidental E.P.E. (CHLO). To calculate our random probabilistic sample size, we used a list of 55 903 patients who entered the ED at the public hospital in Lisbon, Portugal at least once between January 1 and December 31, 2016. When a chosen individual had more than 1 ED admission in the year under study, we chose the last admission according to the date of admission. A 5% margin of error and a 95% confidence interval were used. The representative sample size comprised 382 patients. The data were collected between May and November 2017. The questionnaire was developed using various measurement scales and consisted of 75 questions. It was sent either by mail or e-mail, depending on the respondent’s preference.

For the given analysis, we selected only the main predictors (antecedents) of satisfaction/PQHC that we considered as having statistically significant conditions (*P* ⩽ .05), and some other predictors that had a statistically significant (marginal effects) relationship with satisfaction/PQHC (*P* ⩽ .10). Thus, variables were selected with the consideration that the antecedents (doctors; qualitative perceived waiting time for triage; meeting expectations; information about possible delays; accessibility and availability; qualitative perceived waiting time to be called back by the doctor following examinations and/or tests; and privacy) were significantly correlated with both the mediators (PQHC or satisfaction) and the dependent variable (patients’ complaints). We also determined the effect of the moderators on the relationship between satisfaction or PQHC and patients’ complaints. Variables were selected with the consideration that both satisfaction or PQHC and the moderators (gender; age; number of people in household; level of education; monthly household income; level of satisfaction with life; level of happiness; evaluation of state of health; frequency of ED experiences; duration of symptoms or complaints before going to the ED; existence of illness or chronic health condition; number of chronic illnesses; possession of health insurance) were significantly correlated with the dependent variable (patients’ complaints).

Stepwise multiple linear regression analysis was used to test the mediation and moderation models using the methodology proposed by Baron and Kenny.^11^ In this research, we considered and analyzed different models with regard to satisfaction and PQHC as they have been proven to be distinct concepts.^12^ In addition, we used only qualitative perceived waiting times (evaluated on a scale of 1-10, where 1 means “very long” and 10 means “very fast”) because they had a stronger correlation with satisfaction and PQHC than quantitative perceived waiting times (evaluated with an exact time scale using hours and minutes).

## Results

The descriptive statistics of the main variables used in the models are represented in Table 1.

**Figure 1.**
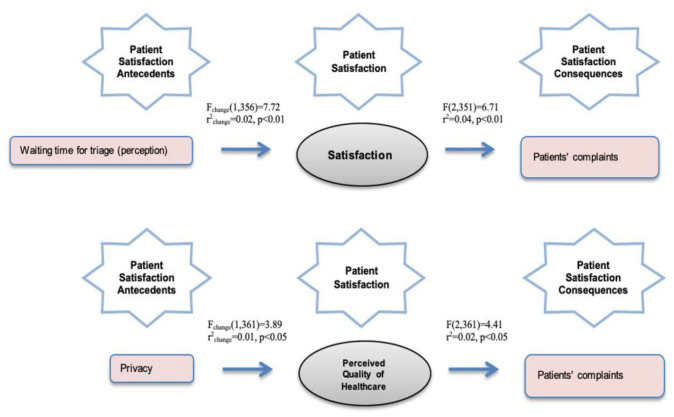
Effect on patients’ complaints.

Only 2 of the mediation models were statistically significant, represented in Figure 1. The models that represent perceived waiting time for triage and privacy show that the contribution of satisfaction and PQHC is 2% and 1% of the explained variance, with statistically significant results (*P* < .01 and *P* < .05).

**Table 1.** Mean, minimum, maximum, and standard deviation related to perceived waiting time for triage, privacy, satisfaction, perceived quality of healthcare, and frequency of ED experiences.

	n		Mean	Min	Max	SD
Perceived waiting time for triage						
Waiting time for triage considering the severity of the condition	362		7.35	1	10	2.37
Privacy						
How privacy was safeguarded	372		7.27	1	10	2.41
Satisfaction						
The level of satisfaction, considering the entire experience in the ED	380		7.10	1	10	2.38
Perceived quality of healthcare						
Overall evaluation of the quality of healthcare	373		7.65	1	10	2.10
	n	%	Mean	Min	Max	SD
Frequency of ED experiences						
Number of times in the ED in 2016			2.21	1	20	2.22
1	121	47.1				
2	75	29.2				
3	24	9.3				
4	16	6.2				
5 or more	21	8.3				
Total	257	100				
Patients’ complaints						
Presentation of verbal or written complaint						
Yes, verbal	30	7.9				
Yes, in writing	14	3.7				
No	335	88.4				
Total	379	100.0				

Without satisfaction or PQHC as a mediator, the effect of perceived waiting time for triage and privacy on complaints is also explained by 2% and 1%. The models without satisfaction and PQHC have an *r* = −.12 correlation level. Adding satisfaction and PQHC in the models reduces the direct correlation level to *r* = −.05 and *r* = .06, showing partial mediation through satisfaction and PQHC. Analyzing the entire models shows that these effects through satisfaction and PQHC are explained by 4% and 2% of the variation, with statistically significant results (*P* < .01 and *P* < .05).

**Figure 2.**
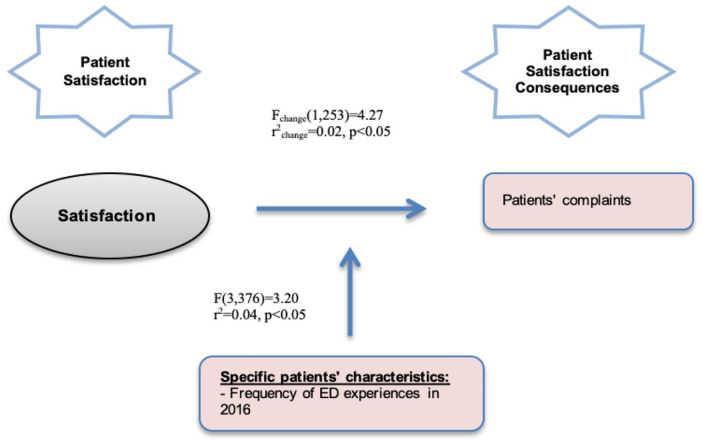
Moderation model.

The moderation models with PQHC were not statistically significant. Only 1 moderation model was statistically significant (with satisfaction). Thus, the contribution of the frequency of ED experiences in the model, represented in Figure 2, is 2% of the explained variance; thus, the effect of satisfaction on complaints is moderated by the frequency of ED experiences by 2%, with statistically significant results (*P* < .05).

Without frequency of ED experiences as a moderator, the effect of satisfaction on complaints is also explained by 2%. We observe different correlation levels between satisfaction and complaints (*r_x_* = .10, *P* < .01), frequency of ED experiences with complaints (*r_m_* = −.15, *P* < .05), and interaction between satisfaction and frequency of ED experiences with complaints (*r_x_*_ *×* *m*_ = −.14, *P* < .01), thus showing a statistically significant moderation effect. Examining the entire model, including this effect of interaction, we observe 4% of the explained variance with statistically significant results (*P* < .05).

The simple slopes analysis shows that the effect of satisfaction on complaints is lower among patients with more frequent ED experiences (*b* = 0.00) than among those with less frequent ED experiences (b=0.05).

## Discussion

Increased efforts have been made to effectively analyze complaints to improve healthcare quality.^13^ Researchers have found that most of the grievances and complaints in the healthcare setting are related to communication problems with hospital employees, followed by patient perceptions that patient safety and medical care have been compromised.^14^ One of the major problems in EDs is overcrowding. Visits from frequent ED users could be a possible reason and explanation for ED crowding that is associated with overstressed healthcare professionals, long waiting times, safety issues, and patient dissatisfaction.^1^

Researchers have pointed out that waiting times^15^ and a lack of privacy and confidentiality^16^ can cause patient complaints, and this is consistent with our results. However, we determined that privacy influences complaints through PQHC, and perceived waiting time for triage influences complaints through satisfaction.

Patient satisfaction may be affected by patient-level characteristics and mental health scores.^17^ Satisfaction level was found to be associated with a range of factors such as sex, age, race, socioeconomic status, education level, and health outcomes.^17^ However, the results in terms of sociodemographic characteristics and clinical variables vary depending on the context of the patient satisfaction measurement. Some researchers did not detect a correlation between the patient satisfaction level and sociodemographic factors; rather, patient satisfaction was found to be associated with perceived state of health.^18^

Instead of using patient characteristics as predictors, we used them as moderators in this study. According to our results, only frequency of ED experiences acts as a moderator in the association between satisfaction and complaints. Satisfaction is less likely to influence complaints among patients with more frequent ED experiences than among those with less frequent ED experiences. Thus, when analyzing complaints, one needs to consider improving satisfaction and/or PQHC, taking into account specific patients’ characteristics.
